# Digital transformation initiatives in higher education institutions: A multivocal literature review

**DOI:** 10.1007/s10639-022-11544-0

**Published:** 2023-03-09

**Authors:** Antonio Fernández, Beatriz Gómez, Kleona Binjaku, Elinda Kajo Meçe

**Affiliations:** 1 Departamento de Informática, Escuela Superior de Ingenierías, Universidad de Almería, 04120 Almería, Spain; 2 Departament de Matemàtiques i Informàtica, Escola Politècnica Superior, Universitat de les Illes Balears, 07122 Palma, Illes Balears Spain; 3 Departament of Computer Engineering, Faculty of Information Technology, Polytechnic University of Tirana, Tirane, Albania

**Keywords:** Higher education, Digital transformation, Digital maturity, Emerging technologies, Smart class, Multivocal literature review

## Abstract

Higher Education Institutions (HEIs) are involved in an evolution to a new model of university called *digital university*. This model implies not only adopting new technologies but also developing an organizational strategic transformation which includes information, processes, human aspects, and more. Because an organization’s digital maturity correlates with the scope of its digital transformation efforts, this study aims to identify digital transformation initiatives (DTI) taken by HEIs, defining the new processes and technologies used to implement them. The main motivation is to have a real and clear vision of how universities are transforming themselves, discovering the most relevant DTI that they have applied and if they are doing it through an integrated plan aligned with a digital strategy, as recommended by experts. We conducted a Multivocal Literature Review, as methodology research, to include both academic and grey literature in the analysis. Main results show that the DTI implemented are primarily focused on *providing a quality and competitive education* (24% of 184 DTI from 39 different universities analyzed). Emerging technologies most frequently used are *advanced analytics* (23%), *cloud* (20%) and *artificial intelligence* (16% of total DTI). We conclude that HEIs are in the first steps to digital maturity as only 1 in 4 have a digital strategy and 56% have launched isolated DTI that are not integrated in a plan and do not have a high strategic return value to the organization.

## Introduction

Higher Education Institutions are involved in an evolution to a new model of university. Cawood (2018) and Wildan Zulfikar et al. (2018) describe some of the drivers of change that will shape the university of the future: increasing competition, digital behavior, changes in work, global mobility, democratization of knowledge and access, continuous learning, and removal of boundaries within industries. Similarly, Bloomberg (2018) and I-SCOOP (n.d.-b) include cultural change, customer-centric changes, and overall business-ecosystem changes. Chapco-Wade (2018) and Prasanna and Choudhury (2013) point out that this necessity is also influenced by the university customers (students) who, due to the era in which they live, have high digital expectations from the university. So, universities are facing a very competitive environment, and they feel the need to leverage new digital capabilities to stay relevant.

Adopting new technologies is not enough for universities, but it implies a great change because, as stated by McCusker and Babington (2015, p. 2), “it means a strategic transformation which includes information, processes, technologies, human aspects, and much more”. According to Hess et al., (2016, p. 3), digital transformation (DT) “involves changes in an organization’s business model caused by the adoption of emerging digital technologies, which result in changes in organizational structures, its products, or its services”. Therefore, new, and emerging technologies present opportunities to improve and transform the university’s business processes to create value through transforming the use of technology into value by attracting more students and improving student and staff experiences to obtain the anticipated benefits and results.

Given the interest raised in recent years by digital transformation in universities, there are few studies that address this issue and that expressly identify the activities carried out in this area and their level of transformation. Our purpose is to discover how universities began their digital transformation, whether by designing a digital strategy and a plan to achieve it before implementing their digital transformation initiatives, by describing all strategic processes which may be empowered by emerging digital technology (Castro-Benavides et al., 2020; Hess et al., 2016), or by launching isolated DT initiatives that are not integrated in a plan (Iyengar et al., 2020). Given that there is no fixed path to achieving digital maturity, this study aims to identify the first initiatives taken by several universities and HEIs, defining the new processes and technologies used to implement such DT initiatives. To that end, we performed a literature review on digital transformation initiatives adopted by universities and HEIs. We selected a multivocal literature review (MLR) as our research method, which is a systematic literature review (SLR) including both formal academic literature and grey literature (e.g., internal reports, strategies or plans, web pages, blog posts, presentations, etc.) (Garousi et al., 2019), because we considered that there is valuable data that may not be in academic articles.

The remainder of the paper is structured as follows: Section 2 presents related work about digital maturity, digital transformation and digitalization applied to universities, followed by Section 3 where we describe the research method used (MLR). In Section 4, all the results of our literature review are explained. Then, in Section 5, we present the main results to our research questions, and in Section 6, we discuss our observations and indicate future research that could be conducted, concluding our study.

## Increasing the digital maturity of a HEI

The greatest pressure on universities to change comes from today's students who demand a flexible, personalized, and real-time educational experience. University vision must place the student experience at its heart (Hoskins, 2018; Yesner, 2020). University leaders interviewed in Stokes et al. (2019) study reinforce the idea that at the core of digital transformation are its customers (students) and that technology is merely the tool (94% of those interviewed think that the most important outcome of digital transformation is improving the student experience and after that, 84% believe in meeting students' demands). Prasanna and Choudhury (2013) indicate that satisfied students are the best advertisement for a university. Moreover, Spies (2017) and Seres et al. (2018) indicate that the goal of digital transformation of higher education is creating new ways of working to deliver services focused on users. Hoskins (2018) and Kane et al. (2017) emphasize that a university can be competitive if it leverages technologies to determine the needs and behavior of students, staff, and researchers and to create the best experience for them.

Accordingly, digital transformation is an organization shift on all levels (Grajek, 2020; Orellana et al., 2019), a creation of a new business model (Powell et al., 2015), a redefinition of the whole business model (Berman, 2012; CISCO, n.d.), a new business model driven by the changes (Minina & Mabrouk, 2019; Wildan Zulfikar et al., 2018). The result of the digital transformation is to enable new processes (Grajek, 2020; Orellana et al., 2019; Powell et al., 2015), to offer a product or service with new features (Limani et al., 2019), through smart integration of digital technologies, processes and competences (I-SCOOP, n.d.-b) and a cultural, organizational, and operational change of an organization (Grajek, 2020; I-SCOOP, n.d.-b; Orellana et al., 2019). The goal is to achieve value proposition, beyond technology (Grajek, 2020; Kane et al., 2015; Orellana et al., 2019), a strategic manner to impact the society adding value to stakeholders (Berman, 2012; CISCO, n.d.), transforming in a strategic way (I-SCOOP, n.d.-b). Schallmo and Williams (2018) reviewed digital transformation definitions used in literature and, regardless of the different terms used, we can define digital transformation in a few words as the process of creating a new strategic business model for the organization, using the latest digital technologies, adding high value for all stakeholders.

Digital transformation usually requires a digital strategy that may include several digitalization projects (i-SCOOP, n.d.; Muro et al., 2017). Digitalization is related to the improvement of business operations (functions, models, processes, activities) or the creation of new revenue streams using digital technologies and data (Chapco-Wade, 2018; i-SCOOP, n.d.; Muro et al., 2017). In fact, Limani et al. (2019) and Muro et al. (2017) claim that at the heart of digitalization is the change in people's jobs through the implementation of digital technologies. In contrast to digital transformation, digitalization is focused on technology as support of the efficient business process. Kane et al. (2015, p. 12) explain that “a digitalization project like automation of processes or training workers to use computers may improve the processes, being faster and more efficient”. Similarly, Bloomberg (2018, p. 6) concludes that “digitalization is about technology while digital transformation is about the customer”. According to Herri et al. (2019), digitalization requires continuous changes while McCusker and Babington (2015) and Faria and Nóvoa (2017) state that a digital transformation strategy is more comprehensive, considering interactions with clients, competitors, and suppliers. Furthermore, digital transformation is a broader concept that includes all aspects of the business, as well as many bridges that are built related to data, processes technology, information, human aspects, and much more (I-SCOOP, n.d.-a).

Instead of digital transformation, Kane (2017, p. 223) prefers to express it as “digital maturity” or the “ability to respond to the emerging digital competitive environment in an appropriate manner”, noting also that the “response is generally learned rather than instinctive”. Brown (2018, p. 12) claims that “digital maturity doesn’t necessarily mean the organization will have all the answers to all digital things, but it does mean that the people within the organization will have the skills and the tools to find those answers quickly and act upon the business needs of the organization rather than just talk about the digital needs”. Thus, reaching digital maturity is a gradual process that unfolds across the organization over time, and no organization can become digitally mature overnight, either. There are different digital developmental stages throughout an organization. Even though different companies may be at different stages of digital maturity, there are always ways that they can continue to grow and adapt to become more digitally mature. It is never too late to begin becoming more digitally mature, and the process is never complete. Kane et al. (2017, p. 6) propose that “achieving digital maturity is an ongoing process; technology shifts and advancements, new business models, and changing market demands will continue to push companies to evolve and grow”, while Brown (2018, p. 2) claims that “you want to fundamentally do things differently using technology. That’s what digital maturity is”.

Leaders in digitally maturing organizations understand that they should take a long view, because the end point of digital change is continually being updated. They should craft strategies that account for what is on the horizon and make the objectives real through technology and processes innovations. D’Antonio (n.d., p. 3) claims that “the path to digital maturity consists of two interweaving processes: the first one is about achieving digital performance, while the second involves the conversion of the organization’s DNA. Characteristics, performance, and essence are necessary for a sustainable digital future”. Therefore, if “digitalization used to be an indicator of business maturity fostering competitive advantage”, then “digital maturity is becoming nowadays a criterion of business health” (Grealou, 2020, p. 7). Thus, “assessing the maturity of the digital initiatives help to understand where the organization overall stands in its industry’s competitive landscape, and to understand how to address shortcomings” (Kane et al., 2017, p. 9).

**Fig. 1:**
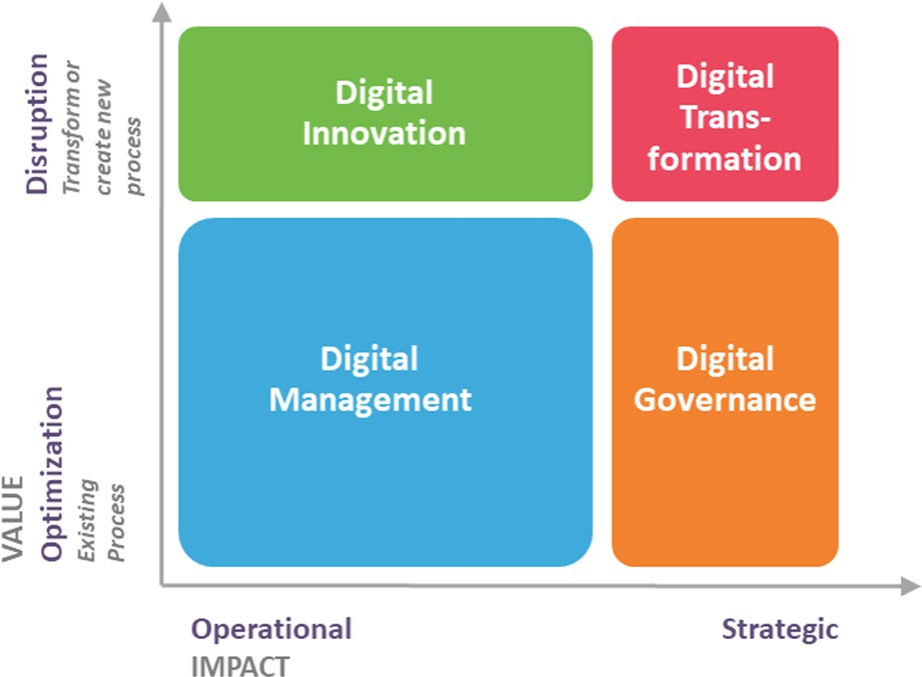
Digital Maturity Model for Universities (md4u) grid (Molina-Carmona et al., 2019)

Assessing IT use maturity at HEIs becomes useful and necessary for two main reasons (Đurek et al., 2018, p. 5): “it can determine how digitally mature a university is based on how ready it is for different IT challenges” and it “can determine what IT areas and fields need improvement”. There are many frameworks for measuring digital maturity in other industries, (e.g., Iyengar et al., 2020; Kane et al., 2017), but very few for HEIs (Doneva et al., 2019; Duarte & Martins, 2011; Đurek et al., 2017; Molina-Carmona et al., 2019; South Australian Government, 2015). Furthermore, Molina-Carmona et al. (2019) propose that digital maturity is a continuous process that grow with the contribution of each digitalization (that could be a digital management or innovation initiative), IT governance and digital transformation initiatives (Fig. 1).

We can then summarize that the digital maturity (DM) of a HEI will grow by adding the implementation of: a) digitalization initiatives (DI), which are technology-driven initiatives whose purpose is to optimize a business operation to achieve a specific benefit, mainly related to cost reduction, or to make processes faster and more efficient; b) IT governance best practices (ITGI), whose goal is to support better strategic decision-making about IT; and c) digital transformation initiatives (DTIs), which are business-driven initiatives whose purpose is to create new business processes that require overall strategic organizational change, using the latest digital technologies, and adding high value to all stakeholders. If HEIs want to increase their digital maturity, they should work in all these areas, but if they want to accelerate their digital maturity, they should invest its efforts in DTIs. Thus, according to Gurumurthy and Schatsky (2019, p. 11), “an organization’s digital maturity correlates with the scope of its digital transformation efforts” and “organizations that are more digitally mature are deriving greater benefit from digital transformation efforts […]. In other words, the more comprehensive and coordinated an organization’s digital transformation efforts are, the more likely it is to be digitally mature”.

Thus, in this paper we analyzed DTIs launched by HEIs to discover whether they are increasing their digital maturity to be as competitive as they need. We focused on whether HEIs defined a digital strategy in advance to be used as a reference to align their implemented DTIs, whether they designed a plan that integrates all the DTIs or they are isolated initiatives, what processes have been digitally transformed first and more frequently by universities, which are the most widely used technologies used by DTIs to implement new processes, and which are the most widely used technologies that have been used to implement each new process.

## Research questions

To achieve our objectives, we formulated five research questions (Table 1).

**Table 1:** Research questions

Number	Question
RQ1	**Is there a digital strategy defined at universities?**
	Our purpose is to discover whether the universities analyzed have an IT strategy (digital strategy, digital transformation strategy, disruptive innovation strategy, a business strategy that includes digital strategy, or similar)
RQ2	**Are digital transformation initiatives (DTIs) integrated into a digital plan or are they isolated initiatives?**
	Our purpose is to discover whether DTIs are integrated into a set of IT initiatives included in a previously designed digital plan or whether they are isolated initiatives launched without a plan and without integration with other IT initiatives
RQ3	**In which strategic processes are universities applying digital transformation?**
	Our purpose is to identify the first strategic processes that universities prefer to transform/create by their digital transformation initiatives to increase the university’s competitiveness
RQ4	**Which are the emerging technologies most used by DTIs to implement new processes?**
	Our purpose is to discover which are the most widely used technologies, focusing on digital transformation initiatives, and excluding digitalization initiatives, i.e., those that increase the strategic value for the university
RQ5	**Which are the emerging technologies that have been used to implement the main new processes?**
	Our purpose is to identify new processes implemented under said technology

In this study, by answering these research questions, we aim to determine the digital transformation level of HEIs, contributing to the awareness of universities and their leaders about the importance of digital transformation and inspiring their leaders to increase the digital maturity. Also, researchers can use the results of this paper to understand where HEIs are in relation to other sectors in the implementation of digital transformation initiatives.

## Methodology

We conducted a Multivocal Literature Review (MLR) (Garousi et al., 2019), which consists of a careful study of the academic literature, similar to a SLR (Kitchenham, 2004; Tocto-Cano et al., 2020), but including grey literature too. We considered grey literature in answering our research questions to provide a greater volume and quality of evidence, including white papers sources, blogs, web pages, university documents published on their websites, university news, etc. as recommended by Garousi et al. (2019). Higher Education Institutions barely published in scientific journals their DTI, they prefer to write white papers or publish their digital transformation success cases at websites. So, if we want to discover the real situation of digital transformation in HEIs, we have to analyze both scientific publications and grey knowledge. To the best of our knowledge, there is no previous MLR for this issue.

**Fig. 2:**
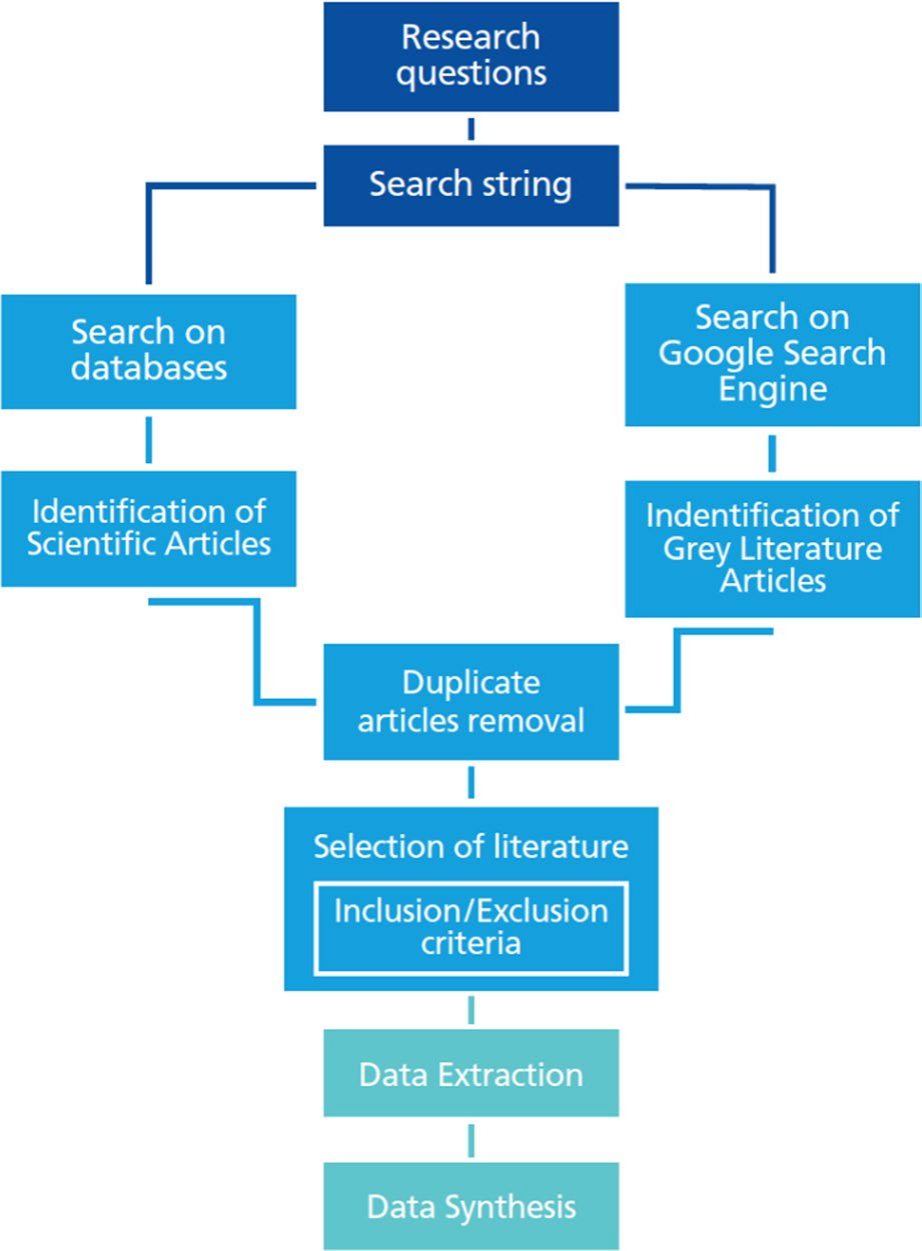
Research methodology phases from Garousi et al. (2019)

To perform our MLR, we followed the model proposed by Garousi et al. (2019) (Fig. 2).

We searched grey literature using the Google search engine and the EDUCAUSE web page specifically, while we searched for academic literature in well-known databases, i.e., Semantic Scholar, ACM Digital Library, IEEE Xplore, ScienceDirect, and Springer. The search strings were formulated to find the related literature according to our research questions; they were: “digital transformation” OR “digital disruption” OR “digital innovation” OR “disruptive technology” OR “innovative services” OR “teaching innovation” OR “business process transformation” AND “university” OR “higher education”.

Using the abovementioned keywords, more than 5000 scientific and grey literature articles were available, but we narrowed the search to the period 2015–2020 because, according to Castro-Benavides et al. (2020), the number of papers on digital transformation in HEIs has increased significantly from 2016, but we also found significant digital transformation initiatives published in 2015. We selected the relevant literature based on the following criteria:

- **Inclusion criteria:** articles published between 2015 and 2020 which gave us information on DT initiatives using the latest emerging IT, that contain the above mentioned search strings, describing digital transformation practices at universities and showing the digital strategy of the university.
- **Exclusion criteria:** articles showing digitalization proposals for universities or those that use the term digital transformation but where the initiative described corresponds to digitalization.

**Fig. 3:**
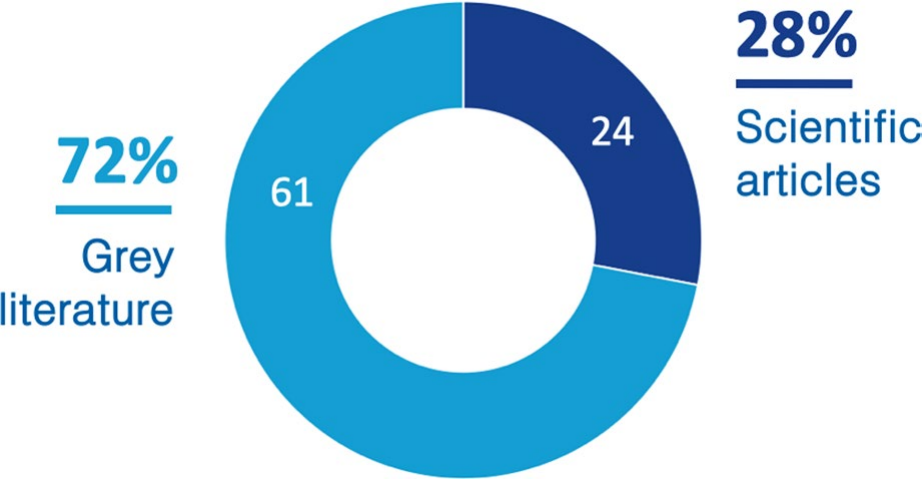
Literature distribution depending on the source (scientific or gray)

The number of articles found using the scientific databases was around 1500; after removing duplicates, around 1200 articles were left. We then went through selection, reading only the abstracts of the articles and considering the keywords and inclusion/exclusion criteria and selected only 120 articles. Reading each of these articles and considering the inclusion and exclusion criteria resulted in 24 academic articles (Fig. 3). In the case of the grey literature, we found around 2500 documents and publications; after removing duplicates and applying inclusion and exclusion criteria, only 61 were relevant to our study.

**Fig. 4:**
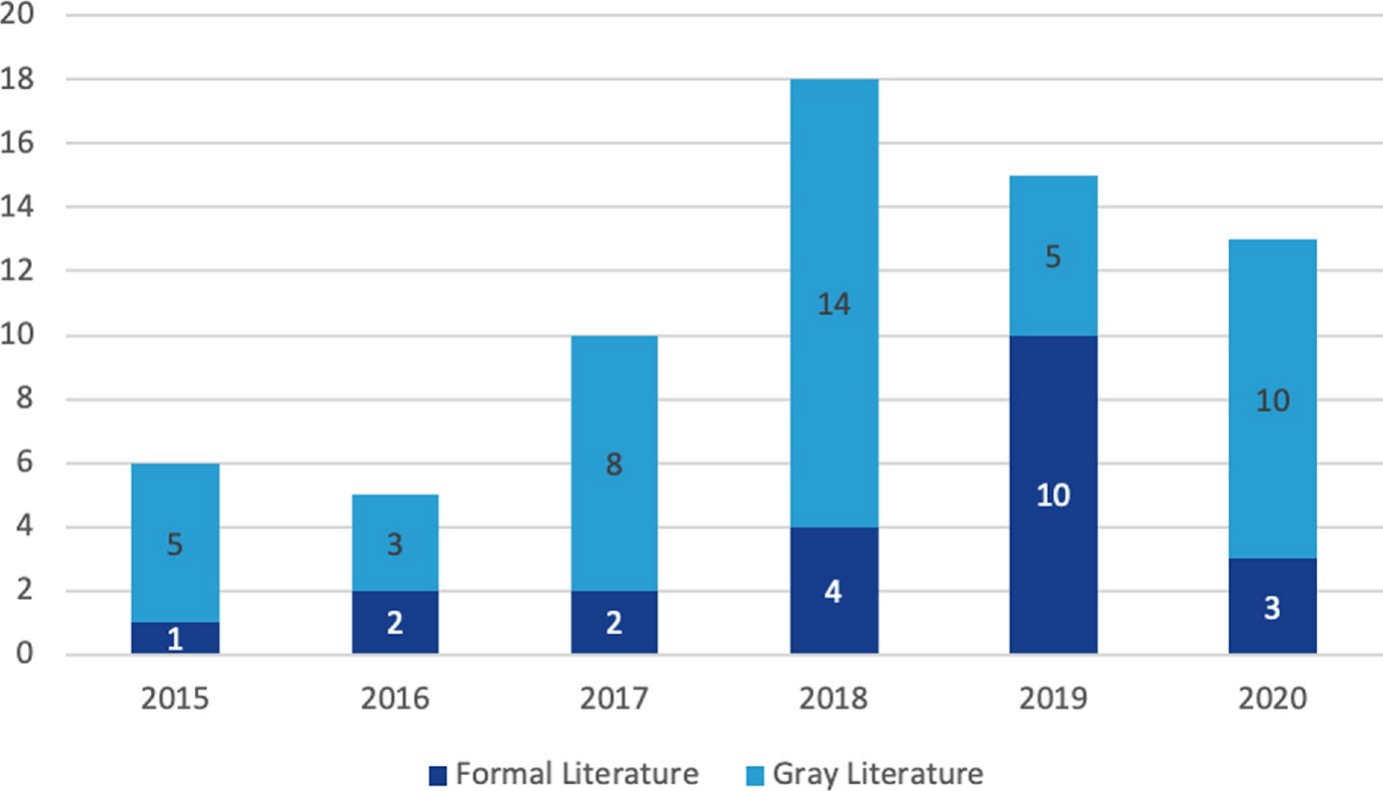
Literature distribution from 2015 included in this research

Figure 4 shows the distribution of the literature from 2015 onwards. Beside 22 articles shown at the figure there are two more that were published before 2015 and beside 45 grey references at the figure there are 16 grey literature articles for which we do not have information about the year of publication.

**Fig. 5:**
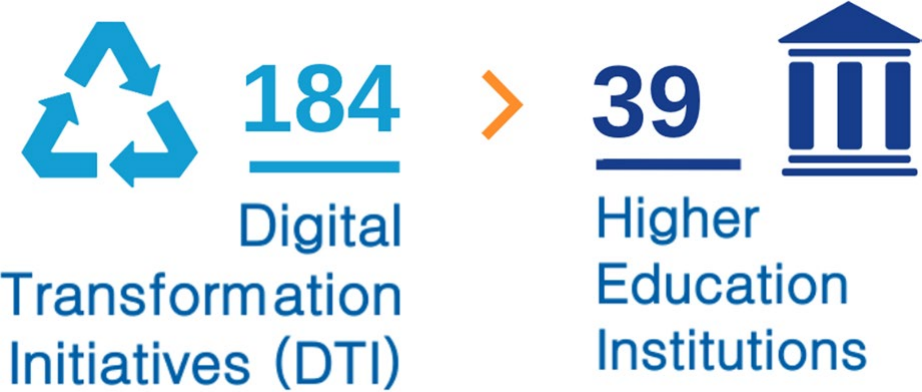
Number of DTI and HEI analyzed in this study

Out of the 24 sources from the formal literature and the 61 sources from the grey literature, we analyzed around 90 universities, found more than 300 IT initiatives, and selected a total of 184 DT initiatives from 39 different universities (Fig. 5) by applying the following criteria:

1. We did not select initiatives that through technology optimize existing processes and initiatives that are focused only on technology, because we do not consider them as digital transformation initiatives, but digitalization initiatives.
2. We included as digital transformation initiatives those that use IT to create new processes, that return a high and strategic value to the university and that consider broader aspects like data, processes, people, etc.

Molina-Carmona et al. (2019) proposed a framework called Digital Maturity for Universities (md4u) that supports the evaluation of the digital maturity of a university in practice. The first level of this framework consists of 7 IT-related strategic challenges that any university should consider increasing its digital maturity and improving its strategic processes. These challenges have been validated by a group of 67 governing body members (half of them Rectors/Presidents and the rest Vice-rectors and CIOs). Thus, we identified DTIs that have created new processes aiming to achieve any of the following strategic challenges:

1. Extend digital skills and culture among the university community.
2. Optimize information security and maintain business continuity.
3. Be competitive thanks to the high quality of services.
4. Offer high quality and competitive education.
5. Satisfy emergent demands of customers (students).
6. Have information and knowledge for optimal decision-making.
7. Achieve the strategic objectives of the university (vision).

## Results

After analyzing the selected academic and grey literature, aiming to answer our research questions, we identified digital transformation initiatives developed in HEIs addressed to achieve the above-mentioned strategic challenges. We studied their integration into digital strategies and plans, and determined the most widely used emerging technologies, both those included in DTIs and those to implement new processes.

### Main digital transformation initiatives in HEIs

**Fig. 6:**
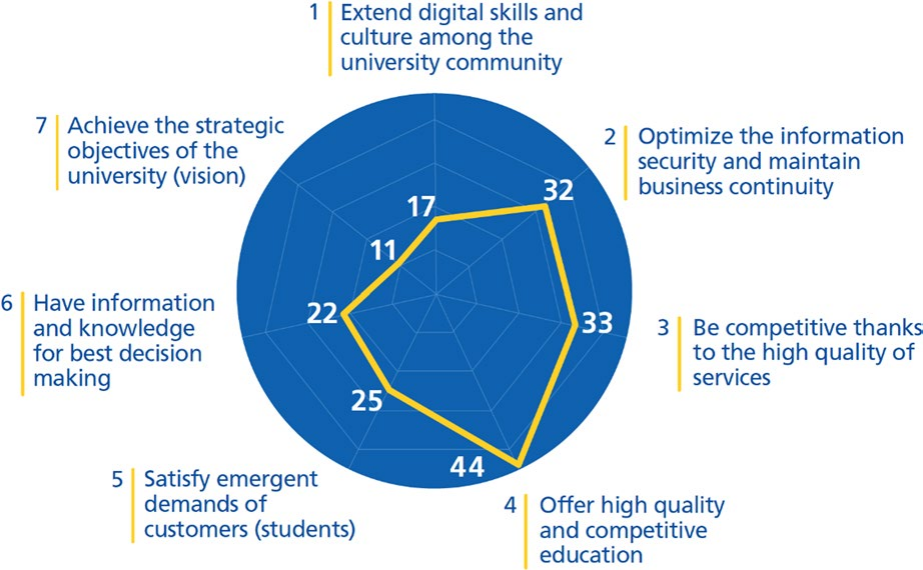
184 DTIs found in universities grouped by challenges of md4u

Many businesses are considering digital transformation as an increasingly important step in their success and survival. Higher education institutions also seek to increase their success and gain an advantage over their competitors. Most of the leaders interviewed in Stokes et al. (2019) study feel threatened by universities that seek to grow their market (77%) and those that seek to enter their geographic area (55%). For this reason, they aim to transform their business models through technology. Organizations need to understand that digital transformation is a relative process and must be tailored specifically to each organization because essentially digital transformation is related to the people and the structure of the organization (Rogers, 2016). Because there is no fixed path, this study aims to identify first successful DTIs taken by 39 HEIs around the world and group them by the 7 challenges (Fig. 6) presented in the md4u model (Fig. 1).

Results show that universities are mainly focused on *providing quality and competitive education* (44 DTIs, i.e., 24% of the total initiatives). The main goal of universities is education and undertaking initiatives toward digital transformation and creating new strategic processes in this direction is a returned value for universities, therefore results are in line with our expectations. There are several models of universities of the future, classified by different sources, and results show an almost uniform distribution of strategic initiatives undertaken towards these models, where life-long learning leads them.

*Provision of quality services, information security and knowledge-based decision-making* grouped around 15% of the initiatives each. Universities are considered a complex business and transforming them strategically should include every aspect of this business.

It was expected that there would be fewer initiatives related to *improving digital skills and spreading the culture of digital transformation in the community* (only 9% of the initiatives). Human behavior is considered as one of the most difficult challenges in any business and the results related to universities confirm this as well.

Digital transformation is mainly about getting a 360º *vision of your customer (students)* and trying to create new processes to *satisfy their emerging demands*, but results show that only 13.5% of the initiatives have been implemented to achieve this goal. It also seems that there are only few initiatives (6% of them) related to *achieving the vision of universities*. This result shows that initiatives tend to be isolated and with no alignment with the university strategy.

Below, we explain in detail the extended processes created by DTIs at universities. The extended processes are categorized according the seven challenges of the md4u framework, and for each one we explain why this process has strategic value for the university.

#### Achieve the strategic objectives of the university (vision)

**Fig. 7:**
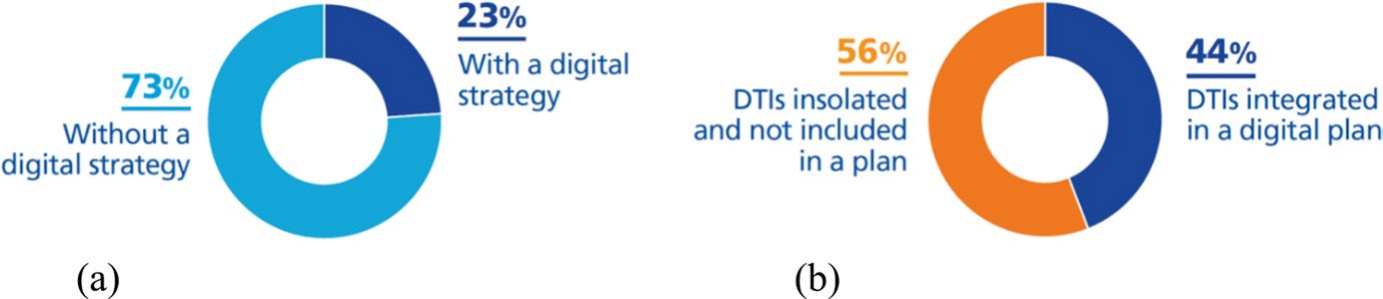
Percentage of universities with a) a digital strategy b) a plan with DTIs integrated

According to our first research question, regarding whether there is an IT strategy defined at the university, we have discovered (Fig. 7a) that 9 universities (23% of the total analyzed) has developed an IT strategy (digital transformation strategy, disruptive innovation strategy, a business strategy that includes digital strategy, or something similar). This means that most of the universities (3 out of 4) have launched their DT initiatives without aligning them with a global business strategy and this could increase the risk of wasting investments on the wrong projects that do not help to return strategic value to the institution.

Regarding our second research question, we have discovered (Fig. 7b) that 17 universities (44% of the total analyzed) based their DT initiatives on a previously designed IT strategy plan. We can highlight that all the universities that have an IT strategy, have an IT plan, so they are included in this category. The rest (56%) are isolated initiatives launched unrelated to a digital strategy or plan, so they do not impact the whole organization and only provide value for a specific department or service.

**Fig. 8:**
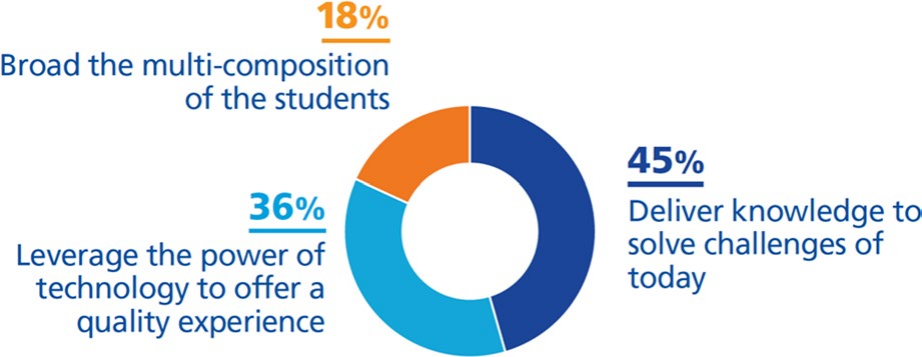
DTIs found relating to the challenge *achieve the strategic objectives of the university (vision)*

Of the total DTIs found, 6% aim to address the first challenge and are divided as shown in Fig. 8:

- ***Deliver knowledge to solve the challenges of today (45%)***. Some of the universities we are referring to aim to provide students with the necessary real knowledge that can be used to solve society's problems. We included universities which have taken initiatives such as providing work-integrated learning as well as intensive research development related to the real challenges of society and in line with the innovation requirements of the economy (Harvard University, 2015, 2018; Queensland University of Technology, 2016; University of York, n.d.). University of York (2018) also aims to be a leader in knowledge creation through applied research in society, while Hogskolen I Oslo Og Akershus (2018) aims to make research transparent and verifiable by linking it to the analysis of large volumes of data. Besides, King’s College London (n.d.) has also taken initiatives not only for quality and impactful research but also for creating collaborations that can be turned into insights or solutions to national challenges and more.
- ***Leverage the power of technology to offer a quality experience (36%)***. Several universities associate their vision with providing a quality experience for students, researchers, and lecturers. To be leaders in this direction Harvard University (2015) and University of Auckland (2020) base decision-making on data, placing the student experience at the heart of this process, while University of Leicester (2018) and Bath Spa University (2020) place technology at the heart of everything to provide innovative research and education. University of Auckland (2020) also includes in its vision the creation of connected experiences and therefore offers shared solutions for data and services and collaborations inside and outside the university.
- ***Broaden the student base (18%)***. Other universities aim to create an educational environment that includes students of all ages, wherever they are. To achieve this, these universities have created special programs that provide flexibility and accessibility while providing data security (Athabasca University, 2018; Lawrence, 2020).

#### Offer high quality and competitive education

According to Spies (2017), HEI leaders who were interviewed see competitive education as a key point of their digital transformation. Offering new forms of quality and competitive education, which improves the student experience, is the challenge where universities are taking more initiatives, a 25% of the total.

**Fig. 9:**
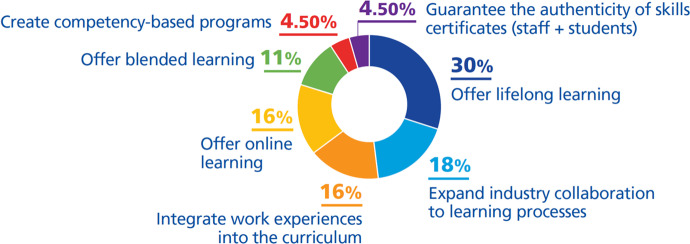
DTIs found relating to the challenge *offer high quality and competitive education*

We have classified these initiatives as follows (Fig. 9):

- ***Offer lifelong learning (30%)***. Universities expand their market and services and become more competitive against competitors or education providers by having continuous learners. Of the universities interviewed by Stokes et al. (2019), 59% answered that the trend that will impact their institution is a decline in students in the traditional age range and an increase in non-traditional student population (working adults, degree completers).
  The former president of Saint Petersburg Electrotechnical University (Minina & Mabrouk, 2019) emphasizes that their challenge is to attract non-traditional students and it seems that most of the universities interviewed feel somewhat confident in their ability to respond to this market trend. Thus, universities have created programs adjusted for new target groups such as professionals with work experience who have completed higher education, practitioners without qualifications but with experience from practice, or persons who are not supported by traditional education (e.g., those who have not finished a higher school or who do not meet the criteria to study a program). The courses are offered in different forms as short programs for mixed groups or in the form of microprograms or nanodegrees (Athabasca University, 2018; Dunagan, 2017; KU Leuven, 2021; Minina & Mabrouk, 2019; Ministry of Education Malaysia (MoE), 2015; Northeastern University, n.d.; Obaid, 2019; RMIT University, 2018; Sandkuhl & Lehmann, 2017; UNIT, 2019; University of Auckland, 2020; University of New South Wales, 2020).
- ***Expand industry collaboration to learning processes (18%)***. There is a need to remove the boundary between university and industry and that can be done through collaboration. Collaboration with industry is very strategic, resulting in an improved student experience, providing resources for the university, guaranteeing job opportunities for students and enrolled adults coming to university to upgrade their knowledge, each of beneficial interest to the university.
  We found that this collaboration has been developed by funding from industry or its involvement in curriculum design (Dunagan, 2017; Ministry of Education Malaysia (MoE), 2015; RMIT University, 2018), in developing products and services (Minina & Mabrouk, 2019; University of Nottingham, 2017), research (Lawrence, 2020), job offers, and training to skill up, targeting adult learners (Dunagan, 2017).
- ***Integrate work experiences into the curriculum (16%)***. Universities are increasingly integrating work experiences into their curriculum based on ‘learn by doing’ practice, to bring students closer to the industry to get them ready for the job market. The learning is oriented and responsive to market needs, following industry developments which for university translates to more interested students (Dunagan, 2017; Fry & Tinson, 2019; King’s College London, n.d.; Powell et al., 2015; Queensland University of Technology, 2016; UNIT, 2019; University of Northampton, 2017).
- ***Offer online learning (16%)***. Going online is one of the challenges of universities today, while also being an objective. Offering online courses increases the flexibility to be accessed by students wherever they are and at any time, so many universities offer online courses in combination with one of the above strategies (i.e., offer continuous learning, expand industry collaboration, integrate work experiences into the curriculum) (California State University San Bernardino, 2018; Dunagan, 2017; Ministry of Education Malaysia (MoE), 2015; Powell et al., 2015; Queensland University of Technology, 2016; Sandkuhl & Lehmann, 2017; University of Auckland, 2020; University of New South Wales, 2020).
- ***Offer blended learning (11%)***. Blended learning offers the flexibility of online learning, keeping the advantage of greater interaction between students and lecturers, also in combination with one of the above strategies (i.e., offer continuous learning, expand industry collaboration, integrate work experiences into the curriculum) (Dunagan, 2017; Fry & Tinson, 2019; King’s College London, n.d.; Queensland University of Technology, 2016; University of Northampton, 2017).
- ***Create competency-based programs (4.5%)***. If teaching is based on competencies rather than contents, the learning can be matched to working skills and the range of interested students will expand since working adults may be interested in upgrading their skills (Dunagan, 2017).
- ***Guarantee the authenticity of skills certificates (staff* + *students) (4.5%)*** University of Leicester (2018) offers internationally recognized student and staff credentials on skills certificates they have gained in training (such as digital skills), through open badges. This is a returned value for the university since it attracts more students and increases collaboration with other universities or third parties (OECD/European Union, 2019).

#### Be competitive thanks to the high quality of services.

Universities aim to improve processes through digital transformation to provide a better experience for users (students, lecturers, researchers, staff, etc.) which is a competitive advantage for the university. Thus, initiatives regarding competitive advantage based on high quality of services represent 17% of the DT initiatives taken by universities.

**Fig. 10:**
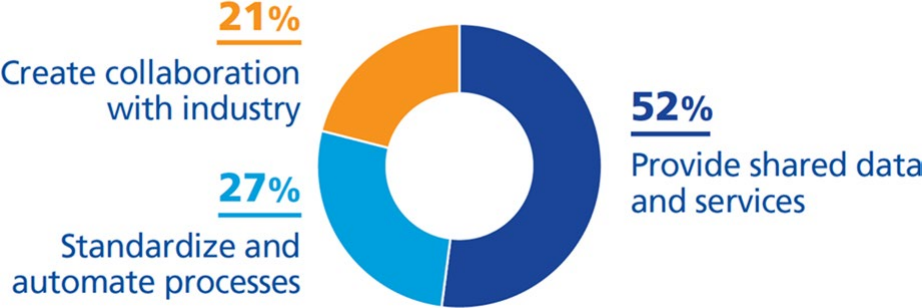
DTIs found relating to the challenge *be competitive thanks to the high quality of services*

We classified them as follows (Fig. 10):

- ***Provide shared data and services (52%)***. Most of the universities are moving towards shared data and services (Athabasca University, 2018; California State University San Bernardino, 2018; Harvard University, 2015, 2018; King’s College London, n.d.; Kuzu, 2020; Lawrence, 2020; Northeastern University, n.d.; Sandkuhl & Lehmann, 2017; SURF, 2018; UNIT, 2019; University of Auckland, 2020; University of Dundee, n.d.; University of Edimburgh, 2017; University of New South Wales, 2020; University of Nottingham, 2017; University of Wisconsin-Stout, 2018; University of York, 2018). They have taken this kind of initiative because sharing data and services leaves greater resources for innovation, enables easily accessible and understandable information and services, increases university visibility, and ensures that data is accurate and timely.
  Thus, according to UNIT (2019) and University of Nottingham (2017), research is moving towards Open Science with publications that can be accessed by everyone, facilitating the work of researchers but also providing transparency and coordination in research. Sharing information and services facilitates data retrieval (Fry & Tinson, 2019; University of Dundee, n.d.); storage and calculations for researchers and administrators (Fry & Tinson, 2019; UNIT, 2019); facilitates documentation management (Sandkuhl & Lehmann, 2017; UNIT, 2019); eliminates the need to duplicate service delivery (University of York, 2018); and removes administrative boundaries (Fry & Tinson, 2019; University of York, 2018).
- ***Standardize and automate processes (27%)***. Other strategic initiatives are the standardization and automation of services (California State University San Bernardino, 2018; Harvard University, 2015, 2018; Ohio University, n.d.; University of New South Wales, 2020). These extended processes add value for universities as researchers, lecturers and administrators will have more free time (Hogskolen I Oslo Og Akershus, 2018; University of Dundee, n.d.; Woods & Lloyd, n.d.), enable the reuse of information (UNIT, 2019) and provide value of money (Lawrence, 2020).
- ***Create collaboration with industry (21%)***. Sharing data and services also enables the establishment of collaborations outside and inside the university with third parties such as industry, local governments, etc., which is another strategic policy of universities because, through them, university can provide resources, high-quality services that maybe cannot provide themselves (Athabasca University, 2018; California State University San Bernardino, 2018; Harvard University, 2015; Northeastern University, n.d.; University of Auckland, 2020).

The results show a considerable number of university DT initiatives to provide high-quality services. Automation, standardization, and shared services improve the productivity and efficiency of the services and user experience, which is a returned value to increase the competitiveness of the university.

#### Optimize information security and maintain business continuity.

For a higher education institution which is taking steps toward digital transformation, setting processes that work accurately and consistently while providing data security is essential. DT initiatives regarding the optimization of information security and maintenance of business continuity represent 16% of the initiatives found.

**Fig. 11:**
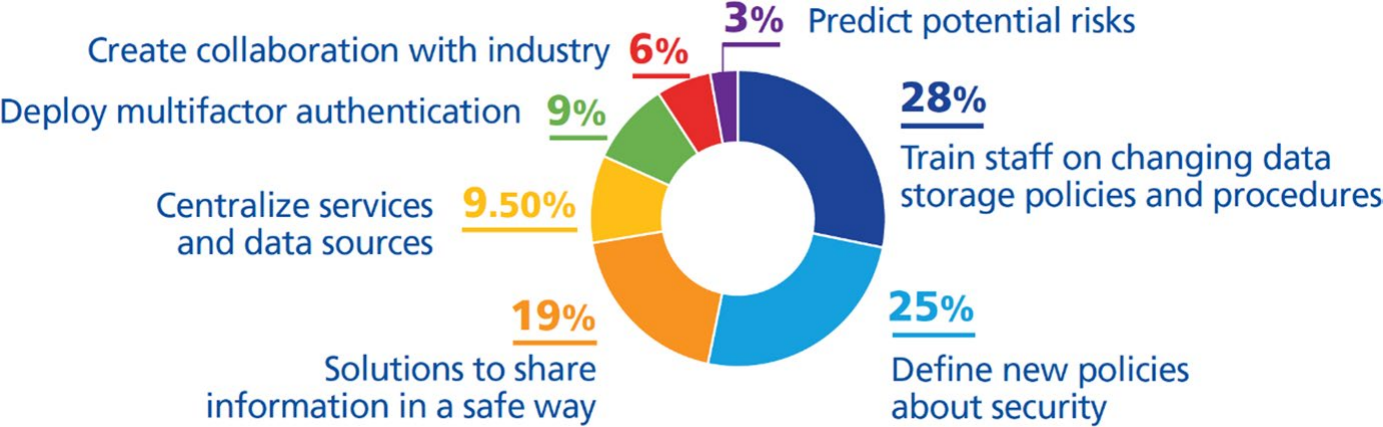
DTIs found relating to the challenge *optimize information security and maintain business continuity*

We have classified them as follows (Fig. 11):

- ***Train staff on changing data storage policies and procedures (28%)***. A very important role in data security is given to user awareness. Universities have taken initiatives to raise awareness among staff, researchers, and students about their role in data security; to train them on changing data storage policies and procedures (Harvard University, 2015, 2018; UNIT, 2019); and to offer advisory services for data security and protection (Athabasca University, 2018; California State University San Bernardino, 2018; North Carolina Department of IT, n.d.; University of Auckland, 2020; University of Leicester, 2018; University of Wisconsin-Stout, 2018; University of York, 2018).
- ***Define new security policies (25%)***. Increased risk for data and services has led to changes in or additions to security policies to ensure safe, efficient, and cost-effective long-term storage (Athabasca University, 2018; SURF, 2018; UNIT, 2019; University of Leicester, 2018; University of York, 2018). The policies of some universities also include the implementation of cybersecurity strategies (North Carolina Department of IT, n.d.; University of Auckland, 2020) or strategies to ensure security and an action plan in case of disasters (Harvard University, 2015, 2018).
- ***Offer solutions to share information safely (19%)***. Shared solution-based infrastructures minimize risks and allow for resource security; security in identity and access management; resilience; portability; disaster recovery; and business continuity solutions, flexibility, ease of access, and lower costs. They also enable mobility and the creation of sufficient space (Athabasca University, 2018; Harvard University, 2018; North Carolina Department of IT, n.d.; UNIT, 2019; University of New South Wales, 2020; University of Wisconsin-Stout, 2018; University of York, 2018).
- ***Centralize services and data sources (9.5%)***. Centralizing services offer easy accessibility, security, off-site access to services and data, and a single source for digital identity (Faria & Nóvoa, 2017; University of Auckland, 2020; University of Leicester, 2018).
- ***Deploy multifactor authentication (9%).*** Controlling access to high risk-data is a key element in information security and universities have taken the initiative to deploy multifactor authentication to secure their sensitive data (Delaney, 2019; Northeastern University, n.d.; University of Wisconsin-Stout, 2018).
- ***Create collaboration with industry (6%)***. In some cases, to manage security independently is difficult for universities, so building partnerships is a strategic decision which is very helpful to design, manage, and continuously improve IT security architecture (Athabasca University, 2018; University of Auckland, 2020).
- ***Predict potential risks (3%)***. Initiatives to predict potential risks have been taken by Athabasca University (2018).

Universities understand that security is a key element that guarantees their business continuity, therefore most of them include security in their strategy. A considerable number of universities have taken strategic initiatives to increase security, not only by exploiting the power of innovative technology, but also by giving an important role to their staff.

#### Have information and knowledge for optimal decision-making.

One of the three core capabilities to navigate digital transformation is informed decision-making (I-SCOOP, n.d.-a). Businesses aim to use data intelligently and to turn it into action. Of the total DTIs found, 14% address having information and knowledge for optimal decision-making.

**Fig. 12:**
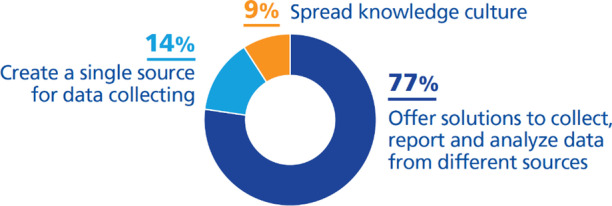
DTIs found relating to the challenge *have information and knowledge for optimal decision-making*

We have divided them as follows (Fig. 12):

- ***Offer solutions to collect, report and analyze data from different sources (77%)***. A great number of higher education institutions are taking initiatives to build an informed decision-making system: using data to adapt the products and services to the preferences of the students and staff (Athabasca University, 2018; Bath Spa University, 2020; Grealou, 2020; Minina & Mabrouk, 2019; University of St. Thomas, n.d.); using data to make decisions about study programs (Hogskolen I Oslo Og Akershus, 2018); using data to offer better educational experiences and services for students, researchers, and staff (Delaney, 2019; Harvard University, 2015; Ohio University, n.d.; University of Leicester, 2018; University of Nottingham, 2017; University of York, 2018; Woods & Lloyd, n.d.); to optimize the infrastructure and resources (Johnson, 2016); and to plan resource allocation (King’s College London, n.d.). Also, they are collecting and analyzing big data volumes and are using them for different purposes (Faria & Nóvoa, 2017; Spies, 2017; UNIT, 2019; University of Dundee, n.d.; University of Edimburgh, 2017).
- ***Create a single source for data collecting (14%)***. In higher education institutions, volumes of data and data sources are growing very fast, so collecting and managing the data is a challenge, but is a critical and essential process to succeed in any digital transformation strategy (I-SCOOP, n.d.-a). Universities have thus taken initiatives to create data collection systems. HEIs are creating shared data sources to reduce the possibility of duplication while providing accurate and real-time data for decision-making (SURF, 2018; UNIT, 2019; University of Dundee, n.d.). They are also taking initiatives to offer capabilities for collecting and reporting that are friendly to everyone because accurate and timely reporting of data increases the speed and accuracy of data-based decision-making (Athabasca University, 2018; University of Edimburgh, 2017).
- ***Spread knowledge culture (9%)***. In this process, building a data-driven culture is essential. HEIs are instilling a data-driven culture, so the decisions at each level of the university are taken based on data (King’s College London, n.d.; San José State University, n.d.).

The results show that a great number of universities are taking initiatives for data-driven decision-making, considering this as the best way to transform their processes. Universities are going toward a data-driven model, based on a knowledge culture and on a single system with a *smart core* that integrates data from different sources.

#### Satisfy emergent demands of customers (students).

More than 90% of UK universities (Fry & Tinson, 2019) and 94% of HEI leaders (Spies, 2017) rank *improving student experience* as the most important benefit of digital transformation. According to Yesner (2020), universities’ mission is to enhance the success of its students by offering them a unified experience. However, initiatives regarding customer satisfaction represent only 12% of the total DT initiatives found.

**Fig. 13:**
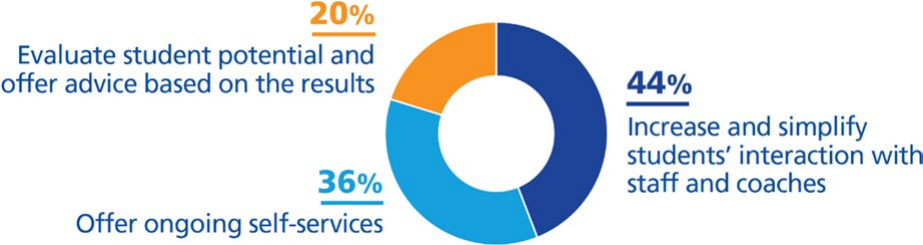
DTIs found relating to the challenge *satisfy emergent demands of customers* (students)

We classified them as follows (Fig. 13):

- ***Increase and simplify students’ interaction with staff and coaches (44%)***. Automated support has not left behind strategies to increase student communication with coaches and academic staff (Harvard University, 2015; Powell et al., 2015; Queensland University of Technology, 2016; Spies, 2017), to support each other through online communities where specific knowledge is disseminated (Delgado-Kloos & Alario-Hoyos, n.d.; Dunagan, 2017; University of New South Wales, 2020) or through the implementation of an Intranet (King’s College London, n.d.; University of Leicester, 2018).
- ***Offer continuous self-service (36%)***. Universities are being strategic by creating self-service units, not only providing support at any time for students, but also by simplifying, automating, and standardizing processes, freeing up time for staff and students (Athabasca University, 2018; California State University San Bernardino, 2018; Hogskolen I Oslo Og Akershus, 2018; King’s College London, n.d.; Manchester Metropolitan University, n.d.; Sandkuhl & Lehmann, 2017; Spies, 2017; University of Edimburgh, 2017; University of York, 2018).
- ***Evaluate student potential and offer tailored advice (20%)***. Exploit the power of data analytics to support students by assessing their potential and advising them on future studies, courses, or books (California State University San Bernardino, 2018; Fry & Tinson, 2019; Obaid, 2019; University of Auckland, 2020; University of Edimburgh, 2017).

The main goal of a digital university seems to be to *improve student experience*, satisfying their emergent demands. But fewer universities than we might expect (only 12%) are achieving it by offering support to the students through self-service units, which aim to increase and simplify the interaction with students; help them to evaluate their potential; and advise them about future courses.

#### Extend digital skills and culture among the university community.

People are the most essential part of an organization. The first step of a higher education institution in transforming its business model thus be through the involvement and training of the institution's staff. According to Herri et al. (2019), a transformation initiative that encounters resistance from those who are part of it may not be successful, as the capacity to adapt to constant change is needed. Queensland University of Technology (2016) and Lawrence (2020) believe that the success of digital transformation relies on the success of the cultural transformation. Only 9% of the DT initiatives found address the extension of digital skills and culture among the university community.

**Fig. 14:**
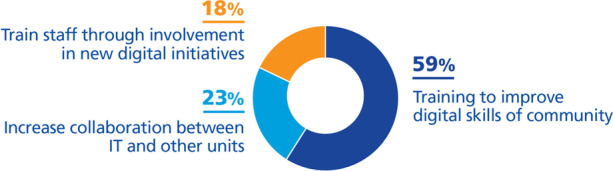
DTIs found relating to the challenge *extend digital skills and culture among the university community*

According to our findings, we have classified them as follows (Fig. 14):

- ***Training to improve digital skills of the community (59%)***. Generally, staff have difficulties accepting new ways of working. According to Fry and Tinson (2019), 70.45% of leaders in UK universities think that the biggest barrier to digital transformation is the culture of the organization, and most of the staff of a university show resistance to transformation and especially to new technologies, thinking that they will be replaced by them. Some universities, for example Athabasca University (2018), just mention that the staff will be trained in new technologies and new ways of working, but without mentioning how this will be carried out. Others, described below, describe the strategic solution to this problem, faced by most university leaders.
  To increase digital capabilities, HEIs are creating spaces for collaboration and real-time interaction and training (Delgado-Kloos & Alario-Hoyos, n.d.; Obaid, 2019; University of Auckland, 2020). Besides, Hogskolen I Oslo Og Akershus (2018) is adapting it to staff needs. Providing guidelines and sharing good practices of upgrading skills are some of the strategies that universities are following to foster a culture of innovation (Queensland University of Technology, 2016; UNIT, 2019; University of Edimburgh, 2017; University of Northampton, 2017; University of Wisconsin-Stout, 2018).
- ***Increase collaboration between IT and other units (23%).*** Universities have established the integration of the IT department with other parts of the organization (Harvard University, 2015; University of Wisconsin-Stout, 2018; University of York, 2018; Woods & Lloyd, n.d.). This way, the role of the IT department shifts from a purely technical role to an advisory one by further disseminating the digital strategy and increasing the demand for customer-focused skills.
- ***Train staff through involvement in new digital initiatives (18%)***. Herri et al. (2019) point out that if leaders have a clear strategic purpose, it is easier to convince staff to understand the importance of each other's contribution to achieving the goals of digital transformation. Likewise, Lawrence (2020) states that success comes from being credible and showing clear leadership. Therefore, to spread the digital culture, Kuzu (2020) claims that involving the staff in the new processes and using emerging technologies more in education make possible an incremental adaptation. Hogskolen I Oslo Og Akershus (2018) is also following the strategy of trial and error to train staff and spread digital culture.

Results show that universities are trying different strategies to deliver digital culture in the community and increase the competences of their staff and students. But a low percentage (only 9%) of DT initiatives are developed to achieve this challenge; universities need to increase the number of initiatives aimed at digital skills and culture to support their digital transformation.

### Emerging technologies most used by digital transformation initiatives.

In the previous sections, we paid attention to identifying new processes that are implemented by digital transformation initiatives of universities, given that the essence of a digital transformation is the creation of a new strategic business model. Digital transformation is based on innovative technologies, so another important aspect is to identify the trends that are being used or that HEIs plan to use to implement DT initiatives.

It should be noted that of 184 DT initiatives that we identified in the previous sections, only 82 have information on the technology used. This is because most of our references deal only with the strategy of the university and do not set out a technological plan.

**Fig. 15:**
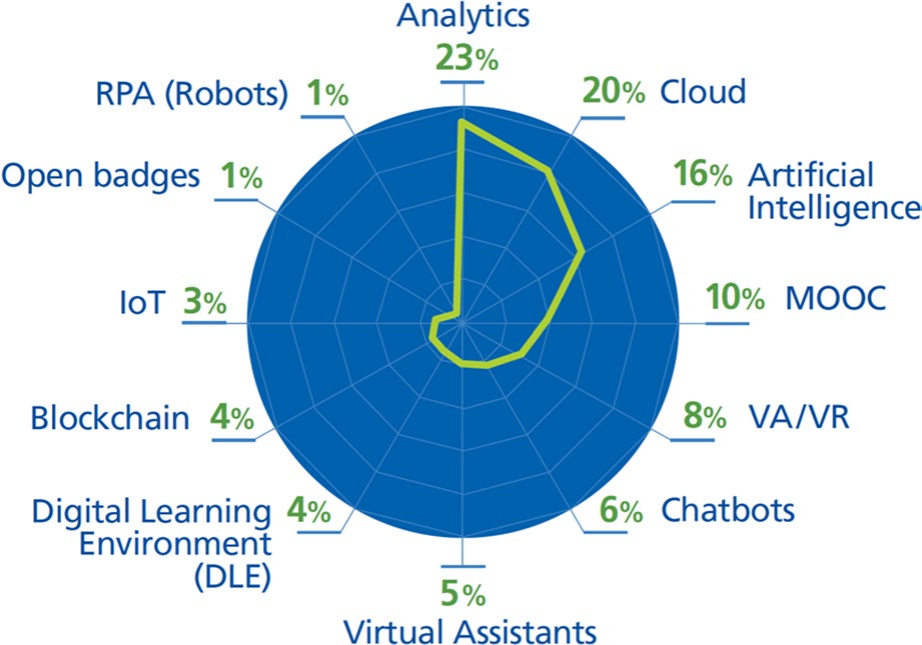
Emerging technologies most used by 82 DTIs found

The most extended technologies found are (Fig. 15):

- **Analytics (used by 23% of the DT initiatives)** are used to monitor and track student progress (Queensland University of Technology, 2016), to identify different groups of students, and to provide personalized teaching (SURF, 2018), experiences, and advice to students and staff (Obaid, 2019; University of Auckland, 2020). The most important application of analytics is supporting decision-making (Athabasca University, 2018; Delaney, 2019; Delgado-Kloos & Alario-Hoyos, n.d.; Faria & Nóvoa, 2017; Fry & Tinson, 2019; Harvard University, 2015; King’s College London, n.d.; University of New South Wales, 2020; University of Northampton, 2017; University of Nottingham, 2017; University of St. Thomas, n.d.). They aim to improve the teaching experience and services by improving support for students, staff, and all stakeholders using analytics on different sources of information such as systems, applications, student feedback, lessons learned from projects, and causes of incidents (University of York, 2018).
- **Cloud technologies (used by 20%)** are primarily used for two purposes: to provide security as well as access anywhere and anytime (California State University San Bernardino, 2018; North Carolina Department of IT, n.d.; Northeastern University, n.d.; University of New South Wales, 2020; University of Wisconsin-Stout, 2018; University of York, 2018); and for disaster recovery by ensuring business continuity (Athabasca University, 2018; Harvard University, 2015; Lawrence, 2020; North Carolina Department of IT, n.d.). In challenge 3 we explained that universities are moving towards a model where data and services are shared. This model relies on cloud technologies that enable the centralization of resources (Faria & Nóvoa, 2017) and provide data and resource sharing without the need for human intervention, duplication of information, removing administrative boundaries, enabling collaborations, and improving the experience of all stakeholders (King’s College London, n.d.; University of Auckland, 2020).
- **Artificial intelligence (16%)** is also essential in data-driven decisions, creating valuable insights about students or staff and then expanding its value to providing personalized experiences to suit everyone's needs (Fry & Tinson, 2019; Obaid, 2019; Spies, 2017; University of Auckland, 2020; University of New South Wales, 2020; Woods & Lloyd, n.d.).
- **MOOC (10%), Digital Learning Environment (4%)** and **open badges (1%)** are the technologies that universities are using in their DT initiatives to provide online teaching. They are using MOOC to offer disruptive education (Athabasca University, 2018; North Carolina Department of IT, n.d.; Obaid, 2019; Powell et al., 2015; Sandkuhl & Lehmann, 2017; Spies, 2017; University of Auckland, 2020; University of Leicester, 2018; University of New South Wales, 2020). Athabasca University (2018) and University of New South Wales (2020) also mention using a Digital Learning Environment (an evolution of LMS), while open badges are only mentioned by the OECD (2019).
- **Virtual and Augmented Reality (9%)** are an emerging trend in education that offer many benefits such as creating digitally enabled learning spaces that offer real-time interaction providing blended learning program (King’s College London, n.d.; Obaid, 2019; Spies, 2017; University of Auckland, 2020; University of New South Wales, 2020).
- **Chatbots (6%), virtual assistants (5%) and RPA (1%)** simplify processes by providing services without the need for human intervention, seamless communication, fast and timely support for students and staff; as well as freeing up time for staff and administrators to deal with more complex problems (California State University San Bernardino, 2018; Hogskolen I Oslo Og Akershus, 2018; Obaid, 2019; Spies, 2017; University of York, 2018).
- **Blockchain (4%),** which is used to store and distribute information securely (Athabasca University, 2018; Spies, 2017), and **IoT (3%)** (Lawrence, 2020; Spies, 2017) are less commonly used trends in DT initiatives.

Technologies with the highest percentage of usage are Analytics (23%), Cloud (20%) and Artificial Intelligence (16%). These results can be explained by the fact that these technologies can be used to implement a wide range of strategic processes created by DT initiatives undertaken by universities. A significant proportion of DT initiatives require decision-making which becomes more strategic if it is based on data rather than centralized. Therefore, analytics and artificial intelligence are used for implementing initiatives in many of the seven challenges, such as providing quality services based on the needs and requirements of all university stakeholders (students, staff), providing personalized education, or monitoring progress of students. AI expands the value chain, including tools for scientific research, enabling chatbots, etc. Cloud is also a technology that has a high percentage of usage (20%) because it serves to implement initiatives such as information security and business continuity, to improve collaborations, or to provide better services. Other technologies such as Virtual and Augmented Reality, MOOC or Virtual Assistants are technologies whose usage in education is emerging. However, the percentage of their usage is lower (5%—10%) because they are relatively new technologies and have high cost and limited content. Finally, RPA, DLE, Blockchain, and IoT are the technologies with the lowest percentage of use (1%—4%) because their direct use in education is limited and they have a moderate potential impact.

### Emerging technologies used to implement new processes

**Fig. 16:**
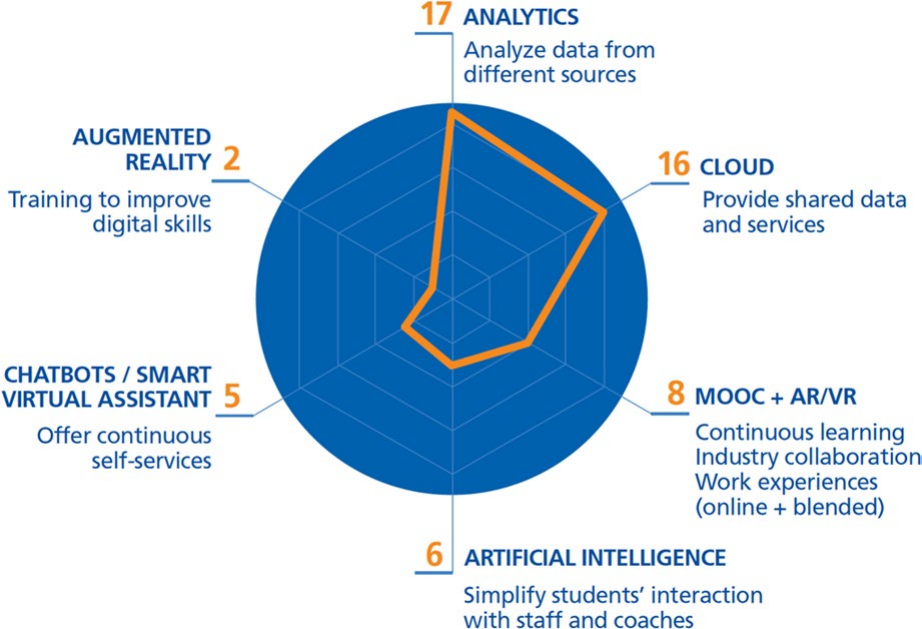
Emerging technologies most used by new processes created by DT initiatives

In this section, Fig. 16 shows which are the technologies most frequently used by the DT initiatives in the review to implement key processes.

*Data analysis* is considered by HEIs as the main process that should be available (17 DTIs, i.e., 9.2% of the total initiatives analyzed); it is implemented using *advanced analytics*. Another important process is *sharing data and services* using *cloud services* (8.7%). The rest are used in far smaller measure: processes relating to *learning and CV experience* (4.4%) using *online and blended learning* (MOOC, AR/VR, etc.), *increasing and simplifying interaction of the students* using *artificial intelligence* (3.3%), *offering continuous self-services* (2.7%) implementing c*hatbots or smart virtual assistants*, and *improving digital skills* through *AR/VR* (1%).

## Discussion

In this paper we analyzed 39 universities and concluded in the following answers to our research questions:

- *RQ1: Is there a digital strategy defined at the university? and RQ2: Are digital transformation initiatives (DTIs) integrated into a digital plan or are they isolated initiatives?*
  We conclude that HEIs are in the first steps of their path to digital maturity because we find that only 1 in 4 universities have designed a digital strategy (aligned with a global business strategy). All the universities that have a digital strategy have implemented a digital plan, and 44% of the total have this kind of plan (digital plan, IT strategy plan, digital transformation plan or similar) as a reference to implement DT initiatives. The rest (56%) have launched isolated DT initiatives that are not integrated between themselves and do not have a high strategic return value to the organization. However, these initiatives are a good way to improve knowledge of the potential of emerging technologies, understanding that they achieve a strategic level impact, and then committing to a comprehensive digital transformation plan.
- *RQ3: In which strategic processes are universities applying digital transformation?*
  By analyzing the DT initiatives found, we identified new strategic processes created to obtain a high and strategic value for the university and emerging technologies used to implement them. The low percentage of DT initiatives taken (only 6%) for the challenge to achieve the strategic objectives of the university (vision) is a proof that most of the universities are in the first steps toward digital transformation, an experimental phase that uses pilot projects and isolated DT initiatives to discover the real potential of the technology trends. HEI leaders see competitive education as a key point of their digital transformation and are working to offer new forms of quality and competitive education, which improves the student experience. This is the challenge where universities are taking more initiatives (25% of the total), dedicated mainly to: offering lifelong learning, expanding industry collaboration, integrating work experiences into the curriculum, offering online and blended learning, creating competency-based programs, and guaranteeing the authenticity of skills certificates (staff and students). Regarding the challenge of providing high-quality services, DT initiatives that have been implemented (18% of the total analyzed) improve the productivity and efficiency of the services through: providing shared data and services, standardizing, and automating processes, and creating collaboration with industry. Universities understand that information security is a key element that guarantees their business continuity, therefore most of them include security in their strategy. A considerable number of DT initiatives (17%) have been taken to increase security, not only by exploiting the power of innovative technology, but also by giving an important role to the people with regard to training staff on changing data storage policies and procedures, defining new policies about security, offering solutions to share information safely, centralizing services and data sources, deploying multifactor authentication, predicting potential risks and creating collaboration with industry.
  One of the three core capabilities to navigate digital transformation is informed decision-making (I-SCOOP, n.d.-a). University leaders aim to use data intelligently and to turn them into action. Of the DT initiatives found, 14% address having information and knowledge to offer solutions to collect, report and analyze data from different sources; create a single source for data collecting; and spread knowledge culture. Universities are going toward a data-driven model, based on a knowledge culture and on a single system with a smart core that integrates data from different sources. The main goal of a digital university seems to be to improve student experience, satisfying their emergent demands. But fewer DT initiatives than we might expect (only 12%) are achieving it, offering support to the students by increasing and simplifying students’ interaction with staff and coaches, offering continuous self-service, and evaluating student potential and offering tailored advice.
  People are the most essential part of an organization; it is thus very important to ensure the involvement and training of the institution's staff. A transformation initiative that encounters resistance from those who are part of it may not be successful, as the capacity to adapt to constant change is needed, but only 9% of the DT initiatives found address the extension of digital skills and culture among the university community, they are dedicated mainly to: training to improve digital skills of community, increasing collaboration between IT and other units, and training staff through involvement in new digital initiatives.
- *RQ4: Which are the emerging technologies most used by DTIs to implement new processes? and RQ5: Which are the emerging technologies that have been used to implement the main new processes?*

In terms of the emerging technologies most used by the 184 DT initiatives analyzed, these are: analytics (23%) and artificial intelligence (16%). These technologies have been used to implement initiatives that require decision-making; to provide quality services based on the needs and requirements of all university stakeholders (students, staff); to provide personalized education; or to monitor progress of students. Cloud technology is also a technology that has a high percentage of usage (20%) because it serves to implement several initiatives, such as information security and business continuity, to improve collaborations or to provide better services. Virtual and Augmented Reality, MOOC or Virtual Assistants are emerging in education and the percentage of their usage is lower (5% - 10%) because they are relatively new technologies and have high cost and limited content. Finally, RPA, DLE, Blockchain, and IoT are the technologies with the lowest percentage of use (1% - 4%) because their direct use in education is limited and they have a moderate potential impact. Offering solutions to analyzed data from different sources using advanced analytics is the process most frequently implemented (17 DTI, which represents 9.2% of the total initiatives analyzed), followed by providing shared data and services using cloud (8.7%).

## Conclusions

In our study, we analyzed scientific and grey literature about the current state of digital transformation in universities through a multi-vocal literature review. We found 24 scientific articles and 61 grey literature references.

The literature proposes investing efforts in different kinds of initiatives to increase the digital maturity of a university: IT governance, digitalization, and digital transformation initiatives. In recent years, universities have made large and successful investments on digitalization. But to accelerate their digital maturation, they need to launch digital transformation initiatives. An organization’s digital maturity correlates with the scope of its digital transformation efforts, and the more comprehensive and coordinated an organization’s digital transformation efforts are, the more likely it is to be digitally mature (Gurumurthy & Schatsky, 2019). So, to accelerate its digital maturity, a HEI should design a digital strategy that commits to digital transformation and develop a plan that integrates all kind of digital initiatives including DT initiatives. All these should be integrated and aligned with the university strategy.

But most of the universities (75%) that we have analyzed does not have a digital strategy, and 56% have launched isolated DT initiatives that are not integrated between themselves and so do not have a high strategic return value to the whole of the organization.

Digital transformation involves the creation of new processes with great and strategic value to the organization. The most important process where universities have applied their DT initiatives (25%) is in improving the quality and offering competitive education, which is the key to attract the students. But they created important processes in relation with services offered (18%), security (17%) and satisfying students demands (12%). The rest of DT initiatives are related to university administration (14% have in focus decision making, 9% to extend the digital skills and culture and 6% to achieve strategic objectives of the university).

Although we tried to involve all universities that have taken initiatives in this regard, there are probably universities that are not included in our study and that could lead to some changes in the results. Not all the universities publish their IT strategic plan and in other cases, we could not access them. Thus, it should be considered as a limitation in our study. The results presented in this paper describe the situation at universities just before the beginning of the COVID-19 pandemic. No doubt the digital situation of universities has changed during the pandemic period, but our contribution could be a good reference for future studies.

Despite these limitations, this study provides some implications for both research on digital transformation and university practice. First, our results should be useful to university leaders in understanding that the digital transformation experiences we presented, and the results of our analysis are so interesting that they can serve as inspiration to begin to increase digital maturity at their HEI and point out the route to continue the process. Second, our results help other researchers to understand how the main goals of the digital transformation proposed in other sectors are implemented or not in HEIs and whether new digital maturity models specific to HEIs can be defined that include the best practices most frequently used. Future analysis can be carried out to understand whether all the changes that have occurred at universities during the pandemic will be consolidated in the future or will be diluted when the health emergency situation is over.

## Data Availability

Not applicable.
